# Exercise and Conjugated Linoleic Acid Supplementation Induce Changes in the Composition of Liver Fatty Acids

**DOI:** 10.3389/fphys.2019.00602

**Published:** 2019-05-15

**Authors:** Adriana Mika, Aleksandra Czumaj, Piotr Stepnowski, Filippo Macaluso, Giulio Spinoso, Rosario Barone, Valentina Di Felice, Tomasz Sledzinski

**Affiliations:** ^1^Department of Pharmaceutical Biochemistry, Faculty of Pharmacy, Medical University of Gdańsk, Gdańsk, Poland; ^2^Department of Environmental Analysis, Faculty of Chemistry, University of Gdańsk, Gdańsk, Poland; ^3^Department of Experimental Biomedicine and Clinical Neurosciences, University of Palermo, Palermo, Italy; ^4^Euro-Mediterranean Institute of Science and Technology, Palermo, Italy; ^5^SMART Engineering Solutions & Technologies Research Center, eCampus University, Novedrate, Italy

**Keywords:** fatty acid, liver, exercise, myokine, conjugated linoleic acid

## Abstract

Exercise and supplementation with conjugated linoleic acid (CLA) are used to reduce body weight and to improve health. Applied together, they may exert a synergistic effect. However, the effects of exercise and CLA supplementation on liver metabolism are poorly understood. The aim of this study was to examine the influence of exercise and CLA supplementation on fatty acids (FA) composition in mouse liver. We analyzed 44 of known FAs of this organ by gas chromatography-mass spectrometry. Our results demonstrated that exercise contributed to a decrease in odd-chain FA and an increase in n-6 polyunsaturated FA contents. In turn, CLA stimulated an increase in branched-chain FAs and n-3 polyunsaturated FAs. Exercise combined with CLA supplementation caused a substantial increase in branched-chain FA content and a slight increase in n-6 polyunsaturated FAs. The elevated liver content of branched-chain FAs after the exercise combined with CLA supplementation, as well as the increase in n-3 polyunsaturated FAs after CLA may be favorable since both these FA groups were previously shown to produce health benefits. However, the expression pattern of enzymes involved in fatty acid synthesis did not match the changes in FA composition. Thus, the mechanism of exercise- and CLA-induced changes in liver FA profile is yet to be established. Also, the consequences of CLA- and/or exercise-induced changes in the composition of liver FAs need to be elucidated.

## Introduction

The hepatic fat composition is determined by a number of processes, e.g., *de novo* lipogenesis, delivery of lipids to the liver from diet or adipose tissue, hepatic lipid oxidation and secretion of intrahepatic lipids to the circulation (Lira et al., 2012; Brouwers et al., 2016). Alterations of these processes are responsible for deposition of the intrahepatic lipids and development of non-alcoholic fatty liver (Brouwers et al., 2016), a condition associated with obesity, insulin resistance and metabolic syndrome (Mika et al., 2017). The hepatic fat composition may also be modified by extrinsic factors, including physical exercise and supplementation with various FAs. While the beneficial effects of exercise on skeletal muscle metabolism have been described in detail, little is known on the influence of physical training on liver metabolism (Brouwers et al., 2016). Previous studies demonstrated that moderate exercise might prevent accumulation of fat in the liver through diminished delivery of lipids to this organ, enhanced hepatic oxidation and increased incorporation of triacylglycerol (TG) into very low-density lipoprotein (VLDL) (Lira et al., 2012; Brouwers et al., 2016; Oh et al., 2017). Conjugated linoleic acid (CLA) has been advertised and promoted as a pro-health and weight loss promoting supplement since the mid-1990s. While supplementation with CLA, indeed, leads to a decrease in body adiposity (Barone et al., 2013; Macaluso et al., 2013), it was also shown to induce hepatic steatosis and to reduce the levels of n-3 and n-6 polyunsaturated FAs (PUFAs) in mouse liver, although these effects seem to be CLA isomer-specific (Vyas et al., 2012). Furthermore, the results of another study involving rats suggest that CLA may, in fact, attenuate liver steatosis (Purushotham et al., 2007).

The aim of the present study was to explore and discuss, using preliminary data, the effects of physical exercise and supplementation with CLA, applied either alone or in combination, on the composition of FAs in mouse liver. Various FAs exert different effects on cell metabolism; for example, n-3 PUFAs are anti-inflammatory fatty acids (FA), whereas saturated FAs (SFAs) induce inflammation (Mika and Sledzinski, 2017). Both physical exercise and supplementation with CLA are used to reduce body weight and to improve health. The effect of both treatments on FA composition in the liver may be vital for the functioning of this organ.

## Materials and Methods

### Animals and Treatment

The study included 32 young (7-week-old) male mice (BALB/cAnNHsd) obtained from Harlan Laboratories S.r.l. (Italy). The mice were maintained under a constant 12:12-h light-dark cycle with free access to food and water. The protocols of all animal experiments were approved by the Committee on the Ethics of Animal Experiments at the University of Palermo (Italy), and compliant with the Guide for the Care and Use of Laboratory Animals of the National Institute of Health (NIH).

The study mice were randomized into four groups, eight animals each: placebo sedentary (PLA-SED), CLA sedentary (CLA-SED), placebo trained (PLA-TR), and CLA trained (CLA-TR). Through the 6-week experiment period, animals from both CLA groups (CLA-SED and CLA-TR) were gavaged with 35 μl d-1 of Tonalin^®^FFA 80 food supplement (Cognis Nutrition and Health, Germany) containing CLA, whereas mice from the placebo groups (PLA-SED and PLA-TR) received 35 μl d-1 of sunflower oil via the same route (Macaluso et al., 2012; Barone et al., 2013, 2017). The gavaged quantities correspond to the 0.5% of food ingested, approximately 4 g (Barone et al., 2013). Tonalin^®^FFA 80 is derived from safflower oil and contains a 50:50 ratio of the active CLA isomers (C18:2 c9, t11 and C18:2 t10, c12).

Animals from both trained groups (PLA-TR and CLA-TR) were trained on a motorized Rota-Rod (Ugo Basile, Biological Research Apparatus, Italy) 5 days/week over a period of 6 weeks. The endurance training began at 3.2 m/min for 5 days/week; during week 1, the mice exercised for 15 min, and the time and speed were systematically increased until week 6, where they were training at 4.8 m/min for 60 min (Di Felice et al., 2007; Barone et al., 2016a). Mice from the sedentary groups (PLA-SED and CLA-SED) did not perform any controlled physical activity throughout the period of the experiment. At 48 h from the end of the last exercise session, all mice were sacrificed by cervical dislocation, and their livers were harvested and weighed (Barone et al., 2016b). The liver was frozen in liquid nitrogen and stored at -80°C for further analyses.

### GCMS Analysis of Fatty Acid Composition in the Liver

The liver samples were extracted in a chloroform-methanol mixture (2:1, v/v) (Folch et al., 1957). Extracted lipids were hydrolyzed with 0.5 M KOH, and the profile of FA methyl esters were determined by gcms using Zebron 5MSi capillary column and electron ionization source, as described previously (Sledzinski et al., 2013).

### Analysis of mRNA Levels

Total mRNA was isolated from 25 mg of mouse liver using GenElute Mammalian Total RNA Miniprep Kit (Sigma-Aldrich, MO, United States), in line with the manufacturer’s instructions. The quality of the resulting RNA was assessed by automated gel electrophoresis (Experion, Bio-Rad Laboratories). Reverse transcription and quantitative real-time PCR were conducted as described previously (Czumaj et al., 2018). The primer sequences are presented in Supplementary Table 1.

### Immunofluorescence and Confocal Analysis

For immunofluorescence, deparaffinised sections were incubated in the antigen unmasking solution (10 mM tri-sodium citrate, 0.05% Tween-20) for 8 min at 75°C, and treated with a blocking solution (3% BSA in PBS) for 30 min. The primary antibody (anti-BCKDHA, diluted 1:50 in PBS, rabbit polyclonal ab90691, Abcam; Cambridge, United Kingdom; anti-BCKDHA (phospho S293), diluted 1:50 in PBS, rabbit polyclonal ab200577, Abcam; anti-ELOVL6, diluted 1:200 in PBS, rabbit polyclonal ab69857, Abcam) was applied and the sections were incubated in a humidified chamber overnight at 4°C. Then, the sections were incubated for 1 h at room temperature with a conjugated secondary antibody (anti-rabbit IgG-FITC antibody produced in goat, diluted 1:100 in PBS, F0382, Sigma-Aldrich, St. Louis, MO, United States). Nuclei were stained with Hoechst Stain Solution (diluted 1:1000 in PBS, Hoechst 33258, Sigma-Aldrich). The slides were treated with PermaFluor Mountant (Thermo Fisher Scientific, Inc., Waltham, MA, United States) and cover slipped. The images were captured using a Leica Confocal Microscope TCS SP8 (Leica Microsystems). The staining intensity was expressed as the mean value pixel intensity (PI) using the software Leica application suite advanced fluorescences software.

### Statistical Analysis

The results are presented as means ± SEM for each study group, if not otherwise specified. The significance of intergroup differences was verified with one-way analysis of variance (ANOVA), or ANOVA on ranks for not normally distributed data, followed by an appropriate *post hoc* test. The threshold of statistical significance for all tests was set at *P* < 0.05. All analyses were carried out with STATISTICA 12 package (StatSoft).

## Results

The results documenting the effects of CLA supplementation and endurance exercise on body weight, skeletal muscle strength, and endurance performance in the study groups have been published elsewhere (Barone et al., 2013). Endurance training or CLA supplementation did not exert a statistically significant effect on the liver weight [PLA-SED: 0.058 ± 0.020 g (liver weight/body weight ± Standard Deviation – SD), PLA-TR: 0.041 ± 0.003 g, CLA-SED: 0.055 ± 0.022 g, CLA-TR: 0.046 ± 0.003 g], however, we observed a trend to decrease of liver weight/body weight ratio after exercise.

### Fatty Acid Composition in Liver After Exercise and CLA Supplementation

The analysis of FA composition in mouse livers demonstrated that physical exercise and supplementation with CLA applied either alone or in combination, induced significant changes in the contents of various FA (Table 1). The training contributed to a significant increase in n-6 PUFAs and a decrease in odd chain FAs (OCFAs), with the latter effect observed solely in PLA-TR mice. Moreover, a tendency for higher values of 18:1/18:0 desaturation index (DI) and lower values of 18:0/16:0 elongation index (EI) was observed in trained animals, but the results did not differ significantly from those for sedentary animals (Table 1). Supplementation with CLA contributed to a significant increase in branched-chain FA (BCFA) and n-3 PUFA contents in sedentary mice. The exposure to both physical exercise and CLA supplementation resulted in a significant increase in BCFA content and a slight increase in n-6 PUFAs. However, the trained and CLA-supplemented mice did not show an increase in n-3 PUFA content, which was observed in PLA-TR animals. The combination of CLA supplementation and exercise contributed also to a non-significant increase in DI values and a significant decrease in EI values (Table 1).

**Table 1:** Fatty acid composition of livers in the studied groups of mice.

The group of fatty acids	Fatty acid	PLA SED (% of total tissue FA ± SEM)	CLA SED (% of total tissue FA ± SEM)	PLA TR (% of total tissue FA ± SEM)	CLA TR (% of total tissue FA ± SEM)
Even-chain saturated FA (ECFA)	C10:0	Traces	0.01 ± 0.00	0.01 ± 0.00	0.01 ± 0.00
	C12:0	0.09 ± 0.03	0.12 ± 0.04	0.09 ± 0.03	0.09 ± 0.01
	C14:0	0.42 ± 0.07	0.35 ± 0.30	0.43 ± 0.02	0.52 ± 0.13
	C16:0	26.77 ± 0.73	26.29 ± 1.70	26.13 ± 0.61	27.36 ± 0.56
	C18:0	10.74 ± 1.23	10.99 ± 0.82	9.46 ± 0.34	8.73 ± 0.66*
	C20:0	0.39 ± 0.03	0.50 ± 0.20	0.28 ± 0.05*	0.30 ± 0.01#
	C22:0	0.20 ± 0.02	0.24 ± 0.08	0.18 ± 0.03	0.17 ± 0.00#
	C24:0	0.09 ± 0.02	0.10 ± 0.02	0.07 ± 0.02	0.06 ± 0.00
	**Total ECFA**	**38.70 ± 1.92**	**38.54 ± 1.62**	**36.64 ± 0.81**	**37.23 ± 1.07**
ODD-chain saturated FA (OCFA)	C11:0	Traces	0.01 ± 0.00	0.01 ± 0.00	0.01 ± 0.00
	C13:0	0.01 ± 0.00	0.02 ± 0.01	0.01 ± 0.00	0.01 ± 0.00
	C15:0	0.11 ± 0.02	0.12 ± 0.02	0.09 ± 0.01	0.14 ± 0.00$#
	C17:0	0.27 ± 0.04	0.29 ± 0.04	0.22 ± 0.02	0.26 ± 0.03
	C19:0	0.10 ± 0.01	0.11 ± 0.02	0.08 ± 0.01*	0.10 ± 0.01
	C21:0	1. ± 0.01	Traces	0.01 ± 0.00	0.01 ± 0.00
	C23:0	2. ± 0.01	0.03 ± 0.01	0.02 ± 0.02	0.03 ± 0.01
	**Total OCFA**	**0.52 ± 0.05**	**0.57 ± 0.08**	**0.43 ± 0.02^∗^**	**0.55 ± 0.04^$^**
Branched-chain saturated FA (BCFA)	*iso* C15:0	0.01 ± 0.00	0.01 ± 0.00	0.01 ± 0.00	0.01 ± 0.00
	*anteiso* C15:0	0.01 ± 0.00	0.01 ± 0.00	0.01 ± 0.00	0.01 ± 0.00
	iso C16:0	0.04 ± 0.01	0.06 ± 0.01$	0.03 ± 0.00*	0.06 ± 0.00#
	iso C17:0	0.04 ± 0.01	0.04 ± 0.01	0.03 ± 0.00	0.05 ± 0.00
	anteiso C17:0	0.03 ± 0.00	0.04 ± 0.0	0.03 ± 0.00	0.13 ± 0.06*$#
	*anteiso* C19:0	0.04 ± 0.01	0.05 ± 0.01	0.05 ± 0.02	0.08 ± 0.00*#
	*anteiso* C21:0	0.01 ± 0.00	0.01 ± 0.00	0.01 ± 0.00	0.01 ± 0.00
	**Total BCFA**	**0.17 ± 0.00**	**0.22 ± 0.03^$^**	**0.16 ± 0.02**	**0.35 ± 0.06**^∗$#^
	CPOA2H	0.02 ± 0.01	0.02 ± 0.01	0.02 ± 0.01	0.02 ± 0.01
Monounsaturated FA (MUFA)	C12:1	Traces	0.04 ± 0.05	0.01 ± 0.01	0.01 ± 0.01
	C14:1	0.02 ± 0.00	0.16 ± 0.23	0.02 ± 0.01	0.03 ± 0.01
	C16:1	2.87 ± 0.40	3.17 ± 0.16	2.59 ± 0.39	3.25 ± 0.55
	C17:1	0.09 ± 0.01	0.12 ± 0.01$	0.09 ± 0.01	0.15 ± 0.00*$#
	C18:1	19.10 ± 1.75	17.10 ± 1.42	20.59 ± 0.90	19.49 ± 1.00
	C19:1	0.01 ± 0.00	0.01 ± 0.01	0.01 ± 0.00	0.02 ± 0.01
	C20:1	0.44 ± 0.03	0.46 ± 0.08	0.50 ± 0.09	0.54 ± 0.05#
	C22:1	0.07 ± 0.01	0.08 ± 0.01	0.07 ± 0.01	0.06 ± 0.00*
	C24:1	0.06 ± 0.03	0.05 ± 0.01	0.05 ± 0.02	0.06 ± 0.01
	**Total MUFA**	**22.67 ± 2.12**	**21.19 ± 1.33**	**23.93 ± 1.12**	**23.58 ± 1.61**
n-3 polyunsaturated FA (n-3 PUFA)	C18:3n-3	0.17 ± 0.02	0.12 ± 0.01$	0.25 ± 0.04*	0.16 ± 0.01*$
	C20:3n-3	0.03 ± 0.01	0.05 ± 0.01$	0.03 ± 0.01	0.04 ± 0.01
	C20:5n-3	0.43 ± 0.06	0.47 ± 0.01	0.44 ± 0.08	0.36 ± 0.03*
	C22:5n-3	0.62 ± 0.09	0.79 ± 0.11	0.60 ± 0.05	0.69 ± 0.08
	C22:6n-3	7.83 ± 0.30	9.34 ± 0.52$	7.33 ± 0.76	7.04 ± 0.45*#
	**Total n-3 PUFA**	**9.07 ± 0.26**	**10.76 ± 0.53^$^**	**8.65 ± 0.77**	**8.29 ± 0.56^∗^**
n-6 polyunsaturated FA (n-6 PUFA)	C16:2n-6	0.01 ± 0.01	0.01 ± 0.00	0.02 ± 0.01	0.03 ± 0.01*
	C18:2n-6	19.41 ± 1.37	18.77 ± 1.01	21.67 ± 1.55	22.21 ± 0.40*#
	C20:2n-6	0.26 ± 0.03	0.34 ± 0.03$	0.23 ± 0.04	0.32 ± 0.01$#
	C20:3n-6	1.03 ± 0.12	1.07 ± 0.15	0.83 ± 0.07	0.85 ± 0.08
	C20:4n-6	7.80 ± 1.07	8.11 ± 1.00	7.14 ± 0.45	6.29 ± 0.35*
	C22:4n-6	0.21 ± 0.04	0.23 ± 0.02	0.16 ± 0.04	0.21 ± 0.04
	C22:5n-6	0.13 ± 0.03	0.16 ± 0.05	0.11 ± 0.03	0.11 ± 0.01
	**Total n-6 PUFA**	**28.86 ± 0.26**	**28.68 ± 0.67**	**30.16 ± 1.02^∗^**	**30.00 ± 0.00^∗#^**
18:1/18:0 desaturation index	1.78 ± 0.38	1.56 ± 0.24	2.17 ± 0.17	2.23 ± 0.28
18:0/16:0 elongation index	0.40 ± 0.04	0.42 ± 0.03	0.36 ± 0.01	0.32 ± 0.02*#

*CPOA2H – Cyclopropaneoctanoic acid 2-hexyl; PLA – placebo, SED – sedentary, TR – trained, ^∗^*p* < 0.05 trained compared to corresponding sedentary group; ^$^*p* < 0.05 CLA treated compared to corresponding placebo group ^#^*p* < 0.05 comparing PLA SED to CLA TR*.

### The Expression of the Enzymes of Fatty Acid Metabolism in Liver After Exercise and CLA Supplementation

To verify if the training- and supplementation-induced differences in liver FA composition resulted from changes in the synthesis, elongation, and desaturation of FA in the liver, we measured the expressions of some enzymes known to be involved in these processes. As the combination of exercise and CLA supplementation exerted the most evident effect on BCFA content, we measured the expression of the alpha subunit of branched-chain alpha-keto acid dehydrogenase enzyme complex (BCKDHA), an enzyme responsible for BCFA synthesis. Moreover, we determined the expression of another two enzymes, stearoyl-CoA desaturase (SCD1), and FA elongase 6 (ELOVL6). Although the analysis demonstrated that both CLA supplementation and physical exercise exerted an effect on BHKDHA, SCD1, and ELOVL6 expressions (Table 2), the pattern of changes in mRNA levels in hepatocytes of trained and supplemented mice did not match with the changes in their BCFA content or 18:1/18:0 and 18:0/16:0 index values (Tables 1, 2).

**Table 2:** Relative mRNA levels of liver enzymes of fatty acid metabolism.

Enzyme	PLA SED	CLA SED	PLA TR	CLA TR
BHKDHA	1.00	6.63 ± 1.45^$^	2.32 ± 0.15	3.81 ± 1.27^#∗^
SCD1	1.00	2.97 ± 0.23^$^	1.83 ± 0.18^∗^	1.76 ± 0.57^#∗^
ELOVL6	1.00	7.68 ± 2.2^$^	3.67 ± 1.2^∗^	1.14 ± 0.47^∗^

*PLA – placebo, SED – sedentary, TR – trained, BHKDHA – alpha subunit of branched-chain alpha-keto acid dehydrogenase enzyme complex, SCD1 – stearoil-CoA desaturase, ELOVL6 – fatty acid elongase 6, ^∗^*p* < 0.05 trained compared to corresponding sedentary group; ^$^*p* < 0.05 CLA compared to corresponding placebo group, ^#^*p* < 0.05 comparing PLA SED to CLA TR*.

**Figure 1:**
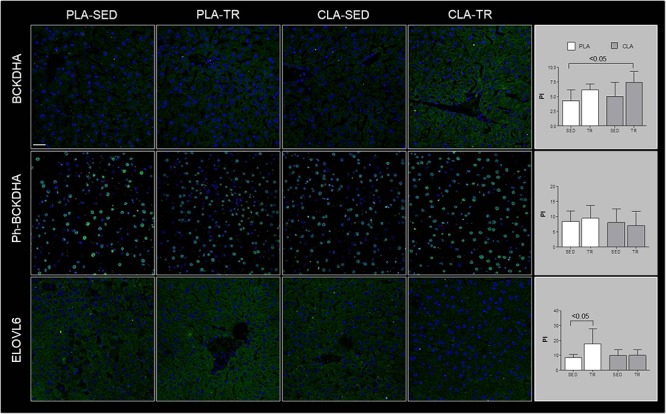
Confocal microscopy analyses. Representative images of immunofluorescence stain for BCKDHA, phospho-BCKDHA and ELOVL6 in liver sections of placebo sedentary (PLA-SED), placebo trained (PLA-TR), CLA sedentary (CLA-SED), and CLA trained (CLA-TR). The staining intensity for BCKDHA, phospho-BCKDHA, and ELOVL6 was expressed as the mean value pixel intensity (PI) using the software Leica application suite advanced fluorescences software. Data are presented as the means ± SD. Bar = 25 μm for all panels.

Confocal microscopy analyses are showed in Figure 1, which indicated that BCKDHA is localized mainly in the mitochondrion matrix and minimally in the nucleus, while phospho-BCKDHA is localized only in the nucleus. The protein level of BCKDHA was significantly higher in CLA-TR group than in PLA-SED, while no differences were observed among the others groups. The endoplasmic reticulum membrane protein, ELOVL6, was significantly higher in PLA-TR group than in PLA-SED, while no differences were observed among the others groups. Thus, changes in protein levels were consistent with the mRNA levels.

## Discussion

This study revealed that physical exercise, supplementation with CLA and combination thereof might induce significant changes in FA composition of mouse liver. The most spectacular change was observed in the case of BCFA content in CLA-TR mice, which was twice as high as in the untreated controls. It should be emphasized that such a significant increase in BCFAs was not observed after either the exercise or the CLA supplementation alone. The effect of exercise and CLA supplementation on BCFA content should probably be considered a favorable finding since elevated levels of FA from this group were previously shown to be associated with some health benefits (Wongtangtintharn et al., 2004, 2005, Ran-Ressler et al., 2011), and their serum levels correlated inversely with hypertriglyceridemia, inflammation and insulin concentration (Mika et al., 2016). Moreover, Garcia Caraballo et al. (2014) found an inverse correlation between liver contents of BCFAs and TG in mice maintained on a high-protein diet, and suggested that the elevated BCFA content may predispose to a decrease in hepatic TG level and lesser steatosis. In mammals, BCFA can be synthesized from branched-chain amino acids (Garcia Caraballo et al., 2014), and the rate-limiting step of these amino acids catabolism is catalyzed by branch chain keto-acid dehydrogenase (BCKDHA) enzyme complex (Crown et al., 2015). However, the patterns of post-treatment changes in BCKDHA mRNA and protein level and BCFA content in our trained and CLA-supplemented mice did not match. We also did not find any differences in phosphorylated (inactive) BCKDHA levels. This implies that the increase in BCFA content observed after exercise and CLA supplementation might have been mediated by other mechanisms (i.e., increased influx of BCFA into hepatocyte).

Another beneficial effect of CLA supplementation documented in our study was an increase in n-3 PUFA content. Previous studies demonstrated that n-3 PUFA might exert anti-inflammatory, insulin-sensitizing, and cardioprotective effects (Mika and Sledzinski, 2017). However, in our study, the beneficial effect of CLA supplementation on n-3 PUFA content was counterbalanced by physical exercise, since we did not find a statistically significant difference in the levels of these FA in supplemented and non-supplemented mice subjected to the Rota-Rod training. In turn, the liver content of n-6 PUFAs increased after training but remained unchanged after CLA supplementation. n-6 PUFAs are precursors of many proinflammatory molecules (Mika and Sledzinski, 2017). Physical exercise is known to attenuate inflammation and oxidative stress in the liver (de Castro et al., 2017). However, the inflammatory response may be triggered by many various factors, and the elevated content of n-6 PUFAs might not be enough to induce inflammation in liver tissues.

We also found that physical exercise resulted in an increase of 18:1/18:0 DI values and in a decrease in 18:0/16:0 EI values. DI and EI are calculated based on the contents of some specific FAs and are considered the measures of SCD1 and ELOVLs activities, respectively (Murakami et al., 2008; Popeijus et al., 2008). However, when we analyzed the profiles of exercise- and supplementation-induced changes in both indices against the patterns of changes in SCD1 mRNA and ELOVL6 mRNA or protein levels, they seemed to be unrelated. This implies that DI and EI values in trained and CLA-supplemented mice did not depend on the expressions of SCD1 and ELOVL6 genes, but were rather influenced by other processes, such as FA oxidation, supply of dietary lipids or release thereof from adipose tissue, and secretion of hepatic lipids to the circulation in the form of lipoproteins.

An important issue that needs to be addressed is the mechanism through which CLA and exercise modulate liver profiles of FAs. A proteomic study showed that some specific CLA isomers might influence the activities of enzymes involved in beta-oxidation and fructose metabolism (Della Casa et al., 2016). Furthermore, exercising muscles release various myokines, and many of them have already been shown to influence the liver metabolism (Carson, 2017). This problem should be elucidated during future research.

In conclusion, this study demonstrated that both supplementation with CLA and physical exercise might induce significant changes in FA profile of the liver. However, the mechanisms and the consequences of CLA- and/or exercise-induced changes in the composition of liver FAs need to be elucidated.

## Ethics Statement

The protocols of all animal experiments were approved by the Committee on the Ethics of Animal Experiments at the University of Palermo (Italy), and compliant with the Guide for the Care and Use of Laboratory Animals of the National Institute of Health (NIH).

## Author Contributions

FM, RB, and VDF performed the animal experiments. AM and PS performed and interpreted GCMS analysis. AC and TS performed and interpreted Real-time PCR analysis. GS performed and interpreted immunofluorescence and confocal analysis. AM, VDF, and TS wrote the manuscript. All authors accepted the final version of manuscript.

## Conflict of Interest Statement

The authors declare that the research was conducted in the absence of any commercial or financial relationships that could be construed as a potential conflict of interest.
