# Supplementary material for: Exercise and Conjugated Linoleic Acid Supplementation Induce Changes in the Composition of Liver Fatty Acids
Source: Front Physiol. 2019 May 15;10:602. doi: 10.3389/fphys.2019.00602 (PMC6529594; doi:10.3389/fphys.2019.00602)
Supplement: Supplementary file 1 [file Table_1.doc]

Table 1. Primers for murine BCKDHA, ELOVL6 and SCD1 genes

| **GENE** | **Forward Primer** | **Reverse Primer** |
| --- | --- | --- |
| BCKDHA | TGGCTAGATCTCACCCCAGCA | AGAGAATGCGGTCCATGGTG |
| ELOVL6 | CATTGGCAGGATCGGTGTCT | CCAGGAAATGGGAGCTGTGT |
| SCD1 | TCATACTGGTTCCCTCCTGC | AGAGCGCTGGTCATGTAGT |
